# Lung Function Impairment and Its Associated Factors in Pulmonary Tuberculosis Patients Upon Treatment Completion in Madurai District, Tamil Nadu: A Cross-Sectional Study

**DOI:** 10.7759/cureus.103941

**Published:** 2026-02-19

**Authors:** Pooranagangadevi Navaneethapandian, Prabhakaran S Sarma, Mahalakshmi Rajendran, Kavumpurathu R Thankappan

**Affiliations:** 1 Public Health, Amrita Institute of Medical Sciences, Kochi, IND; 2 Statistics, Indian Council of Medical Research-National Institute for Research in Tuberculosis, Chennai, IND

**Keywords:** lung function impairment, pulmonary function after tuberculosis, pulmonary function test, pulmonary tuberculosis sequelae, spirometry

## Abstract

Background

While effective anti-tuberculosis treatment (ATT) has led to significant reductions in mortality, concerns persist about the potential for persistent lung damage and impaired pulmonary function among survivors. Data on the prevalence and predictors of lung function impairment (LFI) in post-TB patients in Tamil Nadu are limited. This study aims to evaluate the extent of LFI in pulmonary tuberculosis (PTB) patients upon treatment completion.

Methods

A cross-sectional analytical study was conducted among 132 adult patients aged 18 years and above diagnosed with PTB registered in the government National TB Elimination Program (NTEP) centres, in Madurai district, Tamil Nadu, with smear negative status at completion of standard ATT. Information was obtained on personal habits, respiratory symptoms and co-morbid conditions. A pulmonary function test was done using spirometry. Multiple binary logistic regression analysis was done to find the factors associated with LFI.

Results

Among the patients, 78 (59%; 95% CI (51% to 67%)) showed LFI. The most common abnormality identified was the restrictive pattern, seen in 72 individuals (55%). Patients with a previous history of anti-tuberculosis treatment (adjusted odds ratio (AOR) 2.4; 95% CI 1.0-5.7; p =0.034)), bilateral lung infiltrates (AOR 7.9; 95% CI: 2.7-23.3; p < 0.001), and ≥4 chest X-ray zones (AOR 11.1 CI 2.9-42.3; p <0.001) were more likely to have LFI.

Conclusion

LFI is highly prevalent among PTB patients after treatment completion, with previous TB episodes, radiological severity, and bilateral infiltrates being strong predictors. These findings underscore the necessity of post-treatment pulmonary surveillance, spirometry, and pulmonary rehabilitation to be integrated into TB care frameworks in this population.

## Introduction

Tuberculosis (TB) remains a significant global health challenge, with India carrying a disproportionate burden of the disease [1]. Despite substantial progress in TB control, the long-term consequences of the infection, particularly on respiratory function, are not fully understood [2]. While effective anti-tuberculosis treatment has led to significant reductions in mortality, concerns persist about the potential for persistent lung damage and impaired pulmonary function among survivors, a condition now recognised as post-tuberculosis lung disease (PTLD) [3].

Prevalence estimates for lung impairment after pulmonary tuberculosis (PTB) in India vary widely, ranging from 18% to 87% depending on the study population and assessment methods [4,5]. Recent systematic reviews and meta-analyses in South-East Asia, where India is the leading contributor, report a pooled PTLD prevalence of 57.5% among TB survivors as assessed by spirometry, symptoms, or radiological abnormalities [2]. A restrictive spirometric pattern is often most prevalent, but obstructive and mixed patterns are also common [1,6]. PTLD contributes to a substantial risk of chronic respiratory disability, reduced exercise capacity, and decreased quality of life. It is linked with elevated long-term morbidity and a mortality rate up to 3.8 times higher than in the general population [1,3]. Recurrent TB episodes and incomplete anti-TB therapy further increase the risk and severity of lung impairment [1,7]. Smoking, pollution exposure, nutritional deficits, and socioeconomic status modulate susceptibility and the extent of functional loss. Poor adherence or delays in anti-TB therapy are associated with more severe sequelae.

In two recently published clinical standards, spirometry is considered essential for the evaluation of PTLD at the end of TB treatment [8,9]. Tamil Nadu, a state with a considerable TB burden, provides a valuable context to investigate the impact of TB on lung health. Studies have highlighted the association between TB and respiratory morbidity, but data on the prevalence and predictors of lung function impairment (LFI) in post-TB patients in this region are limited.

This study aims to evaluate the extent of LFI in PTB patients upon treatment completion in Tamil Nadu. By examining factors associated with impaired lung function, this research seeks to contribute to a better understanding of the long-term consequences of TB and inform targeted interventions to improve the quality of life for TB survivors.

Objectives

Primary Objective

The primary objective is to determine the prevalence of pulmonary impairment among PTB patients upon completion of standard anti-tuberculosis treatment in Madurai district, Tamil Nadu.

Secondary Objective

The secondary objective is to identify the clinical and demographic factors associated with pulmonary impairment.

## Materials and methods

A cross-sectional analytical study was done among adult patients aged 18 years and above with a documented history of smear positive pulmonary TB with treatment initiated at selected government National TB Elimination Program (NTEP) centres with smear negative status at completion of standard anti-tuberculosis treatment, declared as “cured” or “treatment completed” under the National TB Program in Madurai district, Tamil Nadu. Those with a history of Concurrent severe respiratory diseases (e.g., chronic obstructive pulmonary disease, asthma), and Uncontrolled Diabetes, Hypertension were excluded.

Assuming the expected proportion of pulmonary function impairment in pulmonary tuberculosis (PTB) cases as 45.4% [10], confidence level of 95%, absolute precision of 9%, and 10% non-response, the sample size of 132 was calculated [10].

All those patients with smear negative status at TB treatment completion were screened for the study, and if found suitable, were referred to the NTEP tertiary care center for the pulmonary function test (PFT) after written informed consent. We recruited participants consecutively for six months till the required sample size was achieved.

Information was gathered about respiratory symptoms, co-morbid illnesses such as diabetes, hypertension, and cardiac issues, and habits like alcoholism and smoking. In addition to recording height, weight, and blood pressure, a general and systemic examination was carried out. Chest X-ray was obtained at the end of treatment to evaluate lung parenchymal damage. The chest X-ray was read by a single radiologist, an independent assessor, who was not aware of the spirometric findings. Specific scoring systems for radiographic zone reading were not used.

PFT was done using Easyone Spirometry in a sitting position as per standard guidelines for performing spirometry. To prevent air leakage, a nose clip was used. About 7-8 spirometry tests were performed for every subject. Acceptable values were those that fell within 5% of the three tests. Forced vital capacity (FVC), forced expiratory volume in one second (FEV1), and the FEV1/FVC ratio were measured. PFT was done immediately on treatment completion, with a median of seven days of treatment completion, with an IQR of 5 to 9 days.

Operational definitions

Airflow obstruction was defined as FEV1/FVC <70% with FVC >80% predicted. Restrictive defects were defined as an FEV1/FVC ratio of >70% with an FVC <80% predicted. Mixed defects were defined as FVC of <80% predicted and an FEV1/FVC ratio of <70%. LFI is defined as the presence of at least one of these three abnormalities [11]. The EasyOne Connect software allows for configuring the "Predicted Reference" to appropriate, region-specific values in the Utilities menu and in this study, we used the predicted reference as established in Indian studies (Joint Indian Chest Society) [11].

Statistical analysis

Collected data were transferred to spreadsheets and IBM SPSS Statistics for Windows, Version 20 (Released 2011; IBM Corp., Armonk, New York, United States) was used for analysis. The parameters of the PFT and participant characteristics were summarised using descriptive statistics. Prevalence of LFI was calculated with 95% confidence intervals. Factors associated with LFI were explored through multivariate analysis (multiple binary logistic regression). A p-value less than 0.05 was considered statistically significant.

## Results

Among the study participants, the majority were in the younger age groups, with the highest proportion of 61 individuals (46.2%) in the 19-30 years category, followed by 34 (25.8%) in the 31-40 group (Table 1). In this cohort of TB patients, 53 individuals (40.2%) were smokers and 50 (37.9%) consumed alcohol. Regarding symptoms, cough was present in 33 participants (25%) and dyspnoea (breathlessness) was reported in 46 (34.8%) (Table 2). Among the participants assessed for lung function, a total of 54 individuals (41%) exhibited normal pulmonary function. However, a larger proportion, 78 individuals (59%), showed LFI, highlighting the impact of TB on pulmonary health. The most common abnormality identified was the restrictive pattern, seen in 72 individuals (55%) (Table 3).

**Table 1:** Sociodemographic and morbidity characteristics of pulmonary TB participants at TB treatment completion (n (%)) BMI: Body mass index; HIV: Human immunodeficiency virus infection. Underweight: BMI less than 18.5; Normal weight: BMI 18.5 to 24.9; Overweight: BMI 25 to 29.9.

Variables	Male (n=101)	Female (n=31)	Total (n=132)
Age group (years)			
19-30	40 (39.6)	21 (67.7)	61 (46.2)
31-40	27 (26.7)	7 (22.6)	34 (25.8)
41-50	18 (17.8)	3 (9.7)	21 (15.9)
51-60	14 (13.9)	Not available	14 (10.6)
61-70	2 (2.0)	Not available	2 (1.5)
BMI (kg/m^2^)			
Underweight	38 (37.6)	4 (12.9)	42 (31.8)
Normal	58 (57.4)	19 (61.3)	77 (58.3)
Overweight	5 (5.0)	8 (25.8)	13 (9.9)
Occupation			
Unemployed	7 (6.9)	18 (58.1)	25 (18.9)
Employed	94 (93.1)	13 (41.9)	107 (81.1)
Diabetes			
No	95 (94.1)	29 (93.5)	124 (93.9)
Yes	6 (5.9)	2 (6.5)	8 (6.1)
HIV Positive			
No	95 (94.1)	28 (90.3)	123 (93.2)
Yes	6 (5.9)	3 (9.7)	9 (6.8)
Prev H/O ATT			
No	53 (52.5)	18 (58.1)	71 (53.8)
Yes	48 (47.5)	13 (41.9)	61 (46.2)
Smear grading			
≤2+	80 (79.2)	25 (80.6)	105 (79.5)
>2+	21 (20.8)	6 (19.4)	27 (20.5)
Cavity in X ray			
No	93 (92.1)	29 (93.5)	122 (92.4)
Yes	8 (7.9)	2 (6.5)	10 (7.6)
Lung Infiltrates			
Bilateral	57 (56.4)	18 (58.1)	75 (56.8)
Unilateral	44 (43.6)	13 (41.9)	57 (43.2)

**Table 2:** Respiratory symptoms and behavioural characteristics of pulmonary TB participants at TB treatment completion (n (%))

Variables	Male (n=101)	Female (n=31)	Total (n=132)
Smoking			
No	48 (47.5)	31 (100.0)	79 (59.8)
Yes	53 (52.5)	Not available	53 (40.2)
Alcohol			
No	51 (50.5)	31 (100.0)	82 (62.1)
Yes	50 (49.5)	Not available	50 (37.9)
Cough			
No	77 (76.2)	22 (71.0)	99 (75.0)
Yes	24 (23.8)	9 (29.0)	33 (25.0)
Dyspnoea			
No	64 (63.4)	22 (71.0)	86 (65.2)
Yes	37 (36.6)	9 (29.0)	46 (34.8)
Expectoration			
No	94 (93.1)	31 (100.0)	125 (94.7)
Yes	7 (6.9)	Not available	7 (5.3)
Hemoptysis			
No	101 (100.0)	30 (96.8)	131 (99.2)
Yes	Not available	1 (3.2)	1 (0.8)
Chest Pain			
No	99 (98.0)	31 (100.0)	130 (98.5)
Yes	2 (2.0)	Not available	2 (1.5)
CXR-Zones			
<4	82 (81.2)	23 (74.2)	105 (79.5)
≥4	19 (18.8)	8 (25.8)	27 (20.5)

**Table 3:** Lung function patterns of the pulmonary TB participants at the end of TB treatment completion (n (%)) LFI: Lung function impairment

Variable	Male (n=101)	Female (n=31)	Total (n=132)
Normal	41 (40.6)	13 (41.9)	54 (40.9)
Restrictive pattern	55 (54.4)	17 (54.8)	72 (54.5)
Obstructive pattern	2 (2.0)	Not available	2 (1.5)
Mixed pattern	3 (3.0)	1 (3.3)	4 (3.1)
Normal	41 (40.6)	13 (41.9)	54 (40.9)
LFI	60 (59.4)	18 (58.1)	78 (59.1)

When evaluating its association with various demographic and clinical variables, there was a significant association of BMI (p = 0.049), with underweight individuals exhibiting a higher prevalence of LFI (30, 71.4%) compared to those with normal or overweight status (48, 53.3%). A significant association was also observed with prior history of anti-TB treatment (p = 0.034), where previously treated individuals had a higher rate of LFI (42, 68.9%) compared to newly diagnosed cases (36, 50.7%). The pattern of lung infiltrates was significant (p = 0.042), with bilateral infiltrates being more strongly associated with LFI (50, 66.7%) than unilateral infiltrates (28, 49.1%) (Table 4). A significant association was observed with radiological extent of disease, specifically the number of chest X-ray (CXR) zones involved. Participants with involvement of ≥4 zones had a significantly higher rate of LFI (21, 77.8%) compared to those with <4 zones (57, 54.3%) (p = 0.027) (Table 5).

**Table 4:** Bivariate analysis of sociodemographic and morbidity characteristics with lung function impairment of pulmonary TB participants at TB treatment completion (N=132) BMI: Body mass index; HIV: Human immunodeficiency virus infection; ATT: Anti-tuberculosis treatment. Underweight: BMI less than 18.5; Normal weight: BMI 18.5 to 24.9; Overweight: BMI 25 to 29.9. *Fisher’s Exact Test

Variables	LFI n (%)	Normal n (%)	p-value
Age group (years)			
19-30	33 (54.1)	28 (45.9)	0.221
31-40	20 (58.8)	14 (41.2)	
41-50	15 (71.4)	6 (28.6)	
51-60	8 (57.1)	6 (42.9)	
61-70	2 (100.0)	Not available	
Sex			
Male	60 (59.4)	41 (40.6)	0.894
Female	18 (58.1)	13 (41.9)	
BMI (kg/m^2^)			
Underweight	30 (71.4)	12 (28.6)	0.049
Normal/Overweight	48 (53.3)	42 (46.71)	
Occupation			
Unemployed	13 (52.0)	12 (48.0)	0.423
Employed	65 (60.7)	42 (39.3)	
Diabetes			
No	74 (59.7)	50 (40.3)	0.716^*^
Yes	4 (50.0)	4 (50.0)	
HIV positive			
No	75 (61.0)	48 (39.0)	0.159^*^
Yes	3 (33.3)	6 (66.7)	
Prev H/O ATT			
No	36 (50.7)	35 (49.3)	0.034
Yes	42 (68.9)	19 (31.1)	
Smear grading			
≤2+	60 (57.1)	45 (42.9)	0.369
>2+	18 (66.7)	9 (33.3)	
Cavity in X-ray			
No	73 (59.8)	49 (40.2)	0.740^*^
Yes	5 (50.0)	5 (50.0)	
Lung infiltrates			
Bilateral	50 (66.7)	25 (33.3)	0.042
Unilateral	28 (49.1)	29 (50.9)	

**Table 5:** Bivariate analysis of respiratory symptoms and behavioural characteristics with lung function impairment of pulmonary TB participants at TB treatment completion (N=132) *Fisher’s Exact Test

Variables	LFI n (%)	Normal n (%)	p-value
Smoking			
No	46 (58.2)	33 (41.8)	0.806
Yes	32 (60.4)	21 (39.6)	
Alcohol			
No	48 (58.5)	34 (41.5)	0.868
Yes	30 (60.0)	20 (40.0)	
Cough			
No	56 (56.6)	43 (43.4)	0.307
Yes	22 (66.7)	11 (33.3)	
Dyspnoea			
No	51 (59.3)	35 (40.7)	0.946
Yes	27 (58.7)	19 (41.3)	
Expectoration			
No	73 (58.4)	52 (41.6)	0.700^*^
Yes	5 (71.4)	2 (28.6)	
Hemoptysis			
No	77 (58.8)	54 (41.2)	1.000^*^
Yes	1 (100.0)	Not available	
Chest Pain			
No	76 (58.5)	54 (41.5)	0.513^*^
Yes	2 (100.0)	Not available	
CXR-Zones			
<4	57 (54.3)	48 (45.7)	0.027
≥4	21 (77.8)	6 (22.2)	

In the multiple binary logistic regression analysis, several variables were examined to identify independent predictors of LFI. A previous history of anti-tuberculosis treatment was significantly associated with LFI, with those having been treated previously showing 2.4 times higher odds of impairment compared to new cases (95% CI: 1.0-5.7; p = 0.034). Importantly, bilateral lung infiltrates were strongly associated with LFI, with nearly 8 times higher odds compared to those with unilateral involvement (adjusted OR = 7.9; 95% CI: 2.7-23.3; p < 0.001). Likewise, patients with ≥4 chest X-ray zones affected had a significantly increased risk of LFI, with an adjusted odds ratio of 11.1 (95% CI: 2.9-42.3; p < 0.001), indicating a strong relationship between radiological disease extent and lung dysfunction (Table 6).

**Table 6:** Multiple binary logistic regression analysis of sociodemographic, respiratory symptoms and behavioral characteristics with lung function impairment of pulmonary TB participants at TB treatment completion (N=132) BMI: Body mass index; HIV: Human immunodeficiency virus infection; ATT: Anti-tuberculosis treatment. Underweight: BMI less than 18.5; Normal weight: BMI 18.5 to 24.9; Overweight: BMI 25 to 29.9.

Variables	Adjusted OR	95% CI	p-value
Age group (in years)			
<30	Ref		
≥30	1.8	0.8-4.3	0.147
BMI (kg/m^2^)			
Normal/Overweight	Ref		
Underweight	2.1	0.8-5.3	0.105
HIV positive			
No	Ref		
Yes	0.762	0.1-3.9	0.745
Previous H/O ATT			
No	Ref		
Yes	2.4	1.0-5.7	0.034
Lung infiltrates			
Unilateral	Ref		
Bilateral	7.9	2.7-23.3	<0.001
Smoking			
No	Ref		
Yes	1.2	0.5-2.8	0.660
CXR zones			
<4	Ref		
≥4	11.1	2.9-42.3	<0.001

## Discussion

This study reveals a high burden of LFI among individuals with a history of PTB, with nearly 59% of participants demonstrating abnormal spirometry, predominantly of the restrictive pattern. These findings are in concordance with several Indian studies, such as one from Puducherry where LFI prevalence was reported at 62.7% [12], and a study from Vellore that showed over 70% of treated PTB patients had spirometric abnormalities [13]. Internationally, similar rates have been documented in a meta-analysis by Allwood et al., who found that up to 68% of post-TB individuals exhibit persistent lung function deficits [14].

The restrictive pattern was the most prevalent, a trend consistently seen in both Indian [12,13] and global settings [14]. This likely reflects residual fibrotic scarring and architectural distortion following TB healing. In our multivariate analysis, history of previous TB treatment, bilateral lung infiltrates, and ≥4 zones of radiographic involvement were significant independent predictors of LFI. These are well-supported by global evidence, such as the systematic review by Meghi et al. that identified radiological extent and disease severity as key correlates of long-term pulmonary impairment [15].

Repeated episodes of TB, particularly those involving retreatment or MDR-TB, are associated with higher odds of fibrosis and bronchiectasis, leading to more severe functional impairment [16,17]. Our study’s finding that prior TB treatment increases the risk of LFI by 2.4 times reinforces this and aligns with Indian studies indicating greater lung damage in re-treatment cases [16].

Radiological severity, especially bilateral involvement and ≥4 zone disease, emerged as robust predictors. This is echoed in both Indian and international literature [2,13,15]. Studies show that higher CXR scores correlate negatively with FEV₁ and FVC [18,19]. An Indian study indicated that each unit increase in the radiological score correlated with a 4-5% decline in lung function measures [2], while Ralph et al. found a direct link between CXR abnormalities and lower diffusion lung capacity for carbon monoxide (DLCO) and total lung capacity (TLC) [19].

Interestingly, respiratory symptoms such as cough, dyspnoea, and expectoration did not show a significant association with LFI in adjusted models. This aligns with evidence suggesting that radiological and spirometric abnormalities may persist even in clinically asymptomatic individuals [15]. Our study also observed higher odds of LFI among underweight individuals, though this was not statistically significant in multivariate analysis. Malnutrition has long been recognised as both a risk factor and a consequence of pulmonary TB in India [20], and its role in recovery and post-TB sequelae merits further exploration.

The high burden of LFI highlights the critical need to incorporate pulmonary rehabilitation (PR) into routine post-TB care. Indian evidence, including the work by Singh et al., demonstrates that structured PR programs yield meaningful gains in FEV₁, six-minute walk distance, and overall health-related quality of life [21]. Internationally, PR is well established as a beneficial intervention for post-TB lung disease (PTLD), with particularly strong relevance for low- and middle-income countries that bear the greatest TB burden [22, 23].

Our study’s strengths include comprehensive data collection integrating clinical, radiographic, and functional assessments, and the use of multiple regression models to identify independent predictors. However, limitations include its cross-sectional nature, limiting causality inference, including near normal patients and lack of baseline spirometric assessment and lack of long-term follow-up or post-rehabilitation outcomes. Additionally, DLCO and high-resolution computed tomography data, which are more sensitive markers of PTLD, were not assessed. Restrictive lung disease is suggested by a reduced FVC on spirometry, but a definitive diagnosis requires confirmation of decreased TLC via plethysmography. Smoking was recorded as binary; residual confounding by cumulative tobacco exposure cannot be excluded in this study.

## Conclusions

Given that India contributes to a quarter of global TB cases, the National TB Elimination Programme must consider integrating post-TB lung assessment, including routine spirometry and rehabilitation referrals, into its guidelines. The WHO’s roadmap for post-TB care also emphasises these components, recommending surveillance of pulmonary function and targeted interventions in high-risk patients.

LFI is highly prevalent among post-TB individuals, with previous TB episodes, radiological severity, and bilateral infiltrates being strong predictors. These findings underscore the necessity of post-treatment pulmonary surveillance, spirometry, and PR to be integrated into TB care frameworks, especially in high-burden countries like India.

## References

[1] Ratnakumar S, Hayward SE, Denneny EK (2025). Post-pulmonary tuberculosis lung function: a systematic review and meta-analysis. Lancet Glob Health.

[2] Ivanova O, Hoffmann VS, Lange C, Hoelscher M, Rachow A (2023). Post-tuberculosis lung impairment: systematic review and meta-analysis of spirometry data from 14 621 people. Eur Respir Rev.

[3] Gai X, Allwood B, Sun Y (2024). Advances in the awareness of tuberculosis-associated chronic obstructive pulmonary disease. Chin Med J Pulm Crit Care Med.

[4] Dhamgaye TM (2022). Post-tuberculosis lung diseases: beyond treatment outcome. J Family Med Prim Care.

[5] Maleche-Obimbo E, Odhiambo MA, Njeri L, Mburu M, Jaoko W, Were F, Graham SM (2022). Magnitude and factors associated with post-tuberculosis lung disease in low- and middle-income countries: a systematic review and meta-analysis. PLOS Glob Public Health.

[6] Meghji J, Auld SC, Bisson GP, Khosa C, Masekela R, Navuluri N, Rachow A (2025). Post-tuberculosis lung disease: towards prevention, diagnosis, and care. Lancet Respir Med.

[7] Woldesemayat EM, Vera JH, Tanner C, Tamiso A, Assefa A, Woldesenbet YM (2025). Lung function of tuberculosis patients after completion of treatment in Sidama, South Ethiopia. Front Med (Lausanne).

[8] Akshaya KT, Fathahudeen A, Kamala R (2025). Proportion of obstructive airway disease among post pulmonary tuberculosis subjects in a tertiary care setting. Int J Med Res Rev.

[9] Seo W, Kim HW, Kim JS, Min J (2024). Long term management of people with post-tuberculosis lung disease. Korean J Intern Med.

[10] Ngahane BH, Nouyep J, Motto MN, Njankouo YM, Wandji A, Endale M, Afane Ze E (2016). Post-tuberculous lung function impairment in a tuberculosis reference clinic in Cameroon. Respir Med.

[11] Aggarwal AN, Agarwal R, Dhooria S (2019). Joint Indian Chest Society-National College of Chest Physicians (India) guidelines for spirometry. Lung India.

[12] Pydipalli M, Chinnakali P, Rajaram M, Sundaram SP, Roy G (2022). Lung function impairment in patients treated for pulmonary tuberculosis and associated factors in Puducherry, South India. Indian J Community Med.

[13] Gupte AN, Paradkar M, Selvaraju S (2019). Assessment of lung function in successfully treated tuberculosis reveals high burden of ventilatory defects and COPD. PLoS One.

[14] Allwood BW, Myer L, Bateman ED (2013). A systematic review of the association between pulmonary tuberculosis and the development of chronic airflow obstruction in adults. Respiration.

[15] Meghji J, Simpson H, Squire SB, Mortimer K (2016). A systematic review of the prevalence and pattern of imaging defined post-TB lung disease. PLoS One.

[16] Dheda K, Limberis JD, Pietersen E (2017). Outcomes, infectiousness, and transmission dynamics of patients with extensively drug-resistant tuberculosis and home-discharged patients with programmatically incurable tuberculosis: a prospective cohort study. Lancet Respir Med.

[17] Pasipanodya JG, Miller TL, Vecino M (2007). Pulmonary impairment after tuberculosis. Chest.

[18] Chung KP, Chen JY, Lee CH (2011). Trends and predictors of changes in pulmonary function after treatment for pulmonary tuberculosis. Clinics (Sao Paulo).

[19] Ralph AP, Kenangalem E, Waramori G (2013). High morbidity during treatment and residual pulmonary disability in pulmonary tuberculosis: under-recognised phenomena. PLoS One.

[20] Padmapriyadarsini C, Shobana M, Lakshmi M, Beena T, Swaminathan S (2016). Undernutrition & tuberculosis in India: situation analysis & the way forward. Indian J Med Res.

[21] Singh SK, Naaraayan A, Acharya P, Menon B, Bansal V, Jesmajian S (2018). Pulmonary rehabilitation in patients with chronic lung impairment from pulmonary tuberculosis. Cureus.

[22] Mbanje C, Kuhn I, Musakwa N (2024). A scoping review of interventions to address TB associated respiratory disability. EClinicalMedicine.

[23] Visca D, Zampogna E, Sotgiu G (2019). Pulmonary rehabilitation is effective in patients with tuberculosis pulmonary sequelae. Eur Respir J.

